# OPTIK: a database for understanding catchment areas to guide mobilization of cancer center assets

**DOI:** 10.1093/database/baaa054

**Published:** 2020-07-28

**Authors:** Dinesh Pal Mudaranthakam, Lisa M Harlan-Williams, Roy A Jensen, Hanluen Kuo, Vandita Garimella, Ronald C Chen, Matthew S Mayo, Hope Krebill

**Affiliations:** 1 Department of Biostatistics & Data Science, University of Kansas Medical Center, Mail Stop 1026, 3901 Rainbow Blvd., Kansas City, KS 66160 USA; 2 The University of Kansas Cancer Center, University of Kansas Medical Center, 3901 Rainbow Blvd., Kansas City, KS 66160 USA; 3 Department of Anatomy and Cell Biology, University of Kansas Medical Center, 3901 Rainbow Blvd., Kansas City, KS 66160 USA; 4 Department of Pathology and Laboratory Medicine, University of Kansas Medical Center, 3901 Rainbow Blvd., Kansas City, KS 66160 USA; 5 Masonic Cancer Alliance, 4350 Shawnee Mission Parkway Suite 1100 Fairway, KS 66205 , USA; 6 Department of Radiation Oncology, University of Kansas Medical Center, 3901 Rainbow Blvd, Kansas City, KS 66160 USA

## Abstract

An increasingly diversified demographic landscape in rural and urban America warrants the attention of The University of Kansas Cancer Center (KU Cancer Center) researchers, clinicians, outreach staff and administrators as the institution assesses ways to reach its expansive, bi-state catchment area. Within the counties of the KU Cancer Center catchment area, patient level and public health data are available and categorized by varying geographic regional boundaries. Multiple data sources and different data collection processes complicate summarizing catchment area data. A curated data warehouse that retrieves and structures the data, with a common denominator, can support meaningful use of the data in a standard and consistent format. The KU Cancer Center built a data warehouse to Organize and Prioritize Trends to Inform KU Cancer Center (OPTIK), which functions to streamline the process of synthesizing data regarding Kansas and Missouri demographics, cancer risk factors and incidence and mortality rates. OPTIK standardizes these diverse data sources to enable analyses of the cancer burden at local, regional and national levels while upholding a strict standard of patient privacy. The OPTIK database enables researchers to use available data and create heat maps and other visualizations to aid in funding proposals, presentations and research activities. Furthermore, using knowledge provided by OPTIK, the KU Cancer Center is able to prioritize action items for research and outreach and more effectively communicate the impact of those efforts.

## Research Support

This study was supported by the National Cancer Institute Cancer Center Support Grant P30CA168524 and used the Biostatistics and Informatics Shared Resource.

## Introduction

Institutions often struggle to understand the evolving populations within their catchment area and are challenged with monitoring how social determinants of health can impact health disparities (1). Identifying appropriate and valid data sources presents real challenges for administrators and researchers (2, 3). In addition, often those in charge of community health improvement may not be familiar with performance measurements to assess improvements (2, 4).

For KU Cancer Center, this challenge is underscored by the institution’s critical role as a National Cancer Institute designated cancer center to define and prioritize cancer research, control and community education and outreach efforts specific to its catchment area (5, 6). The NCI requires that each designated cancer center defines its catchment area and how it serves or intends to serve that catchment area in the research it conducts, the communities it engages and the outreach it performs (6).

The KU Cancer Center serves a patient population that extends across the rural and urban counties of Kansas and western Missouri. This catchment area presents a unique challenge of evaluating cancer needs and issues and therefore warrants a more inclusive approach to the assessment. Catchment area populations can be dynamic. Notably from 2000 to 2010, growth of the rural population in the United States (+2.2 million) slowed, increasing to approximately half of the growth noted between 1990 and 1999 (+4.1 million) (7). However, racial/ethnic minority populations accounted for 82.7% of rural region growth between 2000 and 2010 (7). The decline in overall rural population growth but increase in racial/ethnic minority representation in these areas emphasizes the importance of understanding the heterogeneous population emerging in rural America. The region is diversifying (8) and therefore requires ongoing assessment to understand trends related to health behaviors, cancer incidence and mortality, and access to care in order to strategically focus research and outreach efforts (9). In addition to the evolving rural population within the KU Cancer Center catchment area, there are diverse populations where disparities can be modeled at a census tract level (10). These health disparities are highlighted by two adjacent counties in Kansas. Johnson County, Kansas, ranks number one for the best health outcomes, health behaviors and clinical care in the state. In contrast, the adjacent county of Wyandotte ranks 99 out of 102 for health outcomes, health behaviors at 102 and clinical care at 94, according to the County Health Rankings by Robert Wood Johnson Foundation (11).

The gold standard for any NCI-designated cancer center is to demonstrate impact on their catchment area cancer burden through research, outreach and care (5; 9). Materializing these goals takes shape through focusing research on the cancer burden of the catchment area, promoting cancer prevention and regular screening among populations that experience cancer burden, and catalyzing efforts that contribute to a heightened life expectancy and healthy catchment population (12). However, due to the dynamic nature of a catchment population in rural areas (13), especially regarding changing demographics, it is a challenge for healthcare researchers, clinicians and administrators to prioritize and assess impact of their efforts. Therefore, KU Cancer Center built a data warehouse to Organize and Prioritize Trends to Inform KU Cancer Center (OPTIK). OPTIK helps KU Cancer Center identify cancer issues relevant to its catchment area, strategize research priorities (14), identify cancer health disparities unique to the area and standardize review and prioritization of region-specific factors for research and outreach efforts.

Cancer centers have the onus to contextualize generalized cancer risk factors, such as smoking and obesity, to their target populations to better identify patient needs (15). Data-driven solutions are part of the future of healthcare operations and can be part of the solution to inform outreach and research priorities (16). The KU Cancer Center’s goal is to use data available in OPTIK to summarize the trends and metrics of its bi-state catchment population without having to rely on national trends to influence policy decisions (17, 18). This paper outlines the need for and the process of data consolidation and visualization building at the core of OPTIK’s key functions.

## Materials and methods

### Organizational approach

The KU Cancer Center engaged a catchment area committee comprised of administrators, researchers, community members and state health departments to define the institution’s catchment area, describe social demographics and cancer burden by geographic units, identify cancer disparities and monitor the cancer-pertaining needs of their populations. The catchment area committee identified the need for a locally developed data warehouse to store and organize data specific to our unique bi-state catchment area. The KU Cancer Center’s Biostatistics and Informatics Shared Resource was engaged to build OPTIK, the KU Cancer Center catchment area data warehouse.

Assessing the applicative potential of this data requires a concrete understanding of the KU Cancer Center’s catchment area. However, there are wide swathes of sparsely populated counties in Kansas. To protect privacy, low occurring cancers’ incidence and mortality rates are suppressed at the county level, thus compiling data from geographic areas larger than county level is required for data visualization. Regions in Kansas were selected based on county groupings currently used to aggregate and display data and deploy resources by the Kansas Department of Health and Environment (20). Therefore, eight regions were created in Kansas for OPTIK. The three Missouri regions were created based on the catchment committee’s knowledge of rural versus urban populations, population size of region and proximity.

### Data sources

The process of crafting the OPTIK database began with identifying the key data sources that offered a multidimensional view of the catchment area (19). There are multiple sources for data including public health data, which are often available at either the county or state level, while health system data are available at the individual level. Some data are publicly available, while access to others requires collaboration with organizations and the state health department. The catchment area committee identified and vetted data sources that represent catchment populations’ cancer behavioral risk factors, social determinants of health, screening, incidence and mortality in both Kansas and the 18 counties in western Missouri. Data from a catchment area survey, supported by a Cancer Center Support Grant supplement (P30CA168524), will also be included. Holistically, representing this data set and its diversity in terms of race, ethnicity, rurality and location was prioritized for curation. The selected sources and information gathered from each respective source are outlined in Table 1. Given the KU Cancer Center catchment area spans Kansas and into western Missouri as well; some of the data sources listed in Table 1 are restricted to only one state. As part of the OPTIK build, aggregated data from the various data sources were merged to develop meaningful data visualization that summarizes the information across the KU Cancer Center catchment area. The data for the state of Kansas is then merged with the same data elements of that of the 18 counties under the state of Missouri. Depending upon the risk factors, cancer incidence and mortality information data are retrieved from different data sources as described under Table 1; most of the data is retrieved in the form of flat text files, which are then stored under the SQL (Structured Query Language) Server along with a version number. This server is housed within the University of Kansas Medical Center data center. Once a year, when the data is refreshed, the version number will be updated accordingly.

**Table 1:** OPTIK data sources selected for the initial platform

**Data source**	**Information**	**Extraction process**
Consortium Health System partners	Hospital cancer registry data	Tumor registry data was request per their process
Kansas Cancer Registry	Cancer incidence data	This information is requested on demand from state registry ^***^due to low numbers, we receive this data at region level^***^
Missouri Cancer Registry	Cancer incidence data	This information is requested on demand from state registry
State Cancer Profiles (NCI and CDC)	Cancer mortality data	Data is pulled from the web portal yearly
Kansas Department of Health and Environment	Cancer mortality data	Data related to risk factors such as smoking status, poverty, education and unemployment is retrieved from the website
Missouri Department of Health and Senior Services	Cancer mortality data	Request is submitted to receive this data only on yearly basis
Behavioral Risk Factor Surveillance System (BRFSS)	Cancer screening data, tobacco use	Tobacco usage, mammogram screening information is retrieved from the website
County Health Rankings	Health risk behaviors	Obtained from the website directly
US Census Report	Demographic and socioeconomic data including insurance status	Obtained from the website directly
Health Resources and Services Administration (HRSA)	Healthcare provider shortage designation	Obtained from the website directly
National Immunization Surveys (NIS)	HPV vaccination rates	Obtained from the website directly
Adult obesity	CDC Interactive Atlas	Obtained from the website directly
Centers for Disease Control and Prevention	Vaccination and immunization data	Obtained from the website directly

### Data warehouse design and population

**Figure 1:**
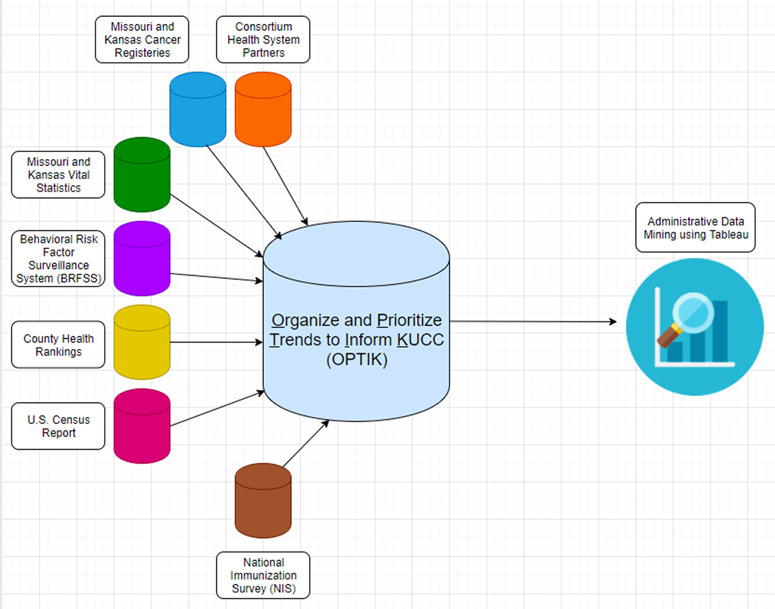
OPTIK data architecture.

Figure 1 reflects a sample of the diverse data sources that were used to create OPTIK, including both publicly available and internal sources representing a heterogeneous spread of region, race, ethnicity and population count. Because the sources include data collected at different geographic levels, data sets were merged at the county level to offer a level of standardization (20). After consolidating sources into the OPTIK database, data are directly plugged into Tableau, a visualization tool that generates heat maps, bar graphs, pie charts and other visual materials (21). Data will be updated approximately once a year, but this process is subject to variation by the database. Data from each of these sources are downloaded in comma-separated text files, which are then uploaded into an SQL database; the SQL database is linked with the Tableau Server version 2018.3. Data scientists use a Tableau Desktop version 2018.3 (64 bit) version to push the new data visualization onto the Tableau server where a wider audience can easily access all the OPTIK-related data visualization. Tableau is utilized to build data visualizations that convey a meaningful trend or pattern to our KU Cancer Center faculty and administration.

## Evaluation

An iterative process was used to define geographic regions larger than county level, validate data sources and improve data visualization (22). The KU Cancer Center Leadership convened key stakeholders and experts to participate in this process. The stakeholders included community research advocates, state health department staff from Kansas and Missouri and state cancer registry directors.

## Results

**Figure 2:**
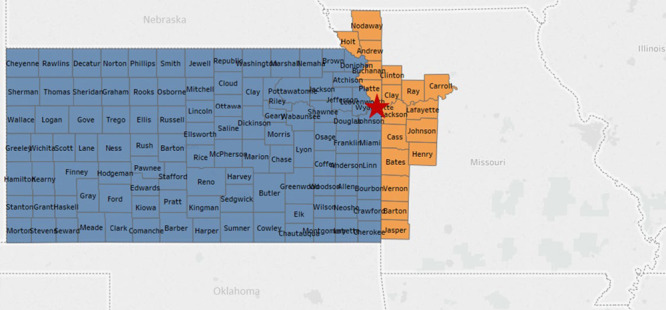
The University of Kansas Cancer Center catchment area. The red star designates the location of the University of Kansas Medical Center in Kansas City, Kansas, Wyandotte County.

**Figure 3:**
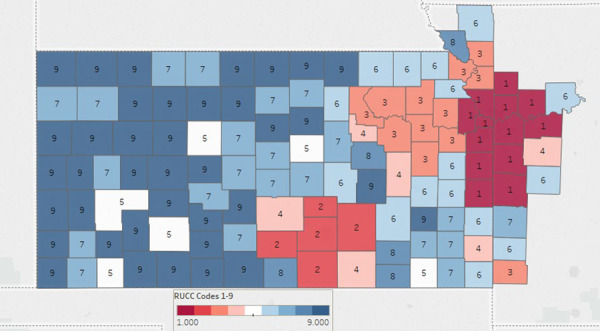
The University of Kansas Cancer Center Catchment Area. Color coding classifies counties by population density. Urban (150.0 persons per square mile (ppsm) or more), semi-urban (40–149.9 ppsm), densely settled rural (20–39.9 ppsm), rural (6–19.9 ppsm) and frontier (<6 ppsm).

The KU Cancer Center’s catchment area includes 105 counties in Kansas and 18 counties in Missouri, representing over 4.4 million people (Figure 2). The 123 Kansas and Missouri counties that constitute the KU Cancer Center catchment area include 93 counties (76%) that are considered rural by rural urban continuum codes (RUCC) 4–9 (Figure 3), (23). Table 2 provides additional insight into the KU Cancer Center catchment area population. United States census numbers indicate the catchment area population is 86.2% White, 8.1% Black or African American and 10% Hispanic, compared to national demographics of 77% White, 13% Black Or African American and 18% Hispanic (24). Using RUCC, nearly 1 million individuals live in rural areas (Table 2).

**Table 2:** The University of Kansas Cancer Center Catchment Area. Population numbers are categorized by race, ethnicity and rural–urban continuum codes

Racial categories	Total	Rural population (RUCC 4–9)	Urban population (RUCC 1–3)
**American Indian**	45 266 (1%)	13 946 (1.3%)	31 319 (0.9%)
**Asian**	109 004 (2.5%)	13 843 (1.3%)	95 161 (2.9%)
**Native Hawaiian or other Pacific Islander**	7604 (0.2%)	1498 (0.1%)	6107 (0.2%)
**Black or African American**	357 186 (8.1%)	28 071 (2.5%)	329 115 (10%)
**White**	3 793 596 (86.2%)	1 027 143 (93.2%)	2 766 452 (83.8%)
**Other**	89 927 (2%)	17 988 (1.6%)	71 938 (2.2%)
**Total**	4 402 583 (100%)	1 102 490 (100%)	3 300 093 (100%)
**Ethnicity categories**			
**Hispanic or Latino**	439 034 (10%)	127 344 (11.6%)	311 690 (9.4%)
**Not Hispanic or Latino**	3 963 549 (90%)	975 146 (88.4%)	2 988 403 (90.6%)

**Figure 4:**
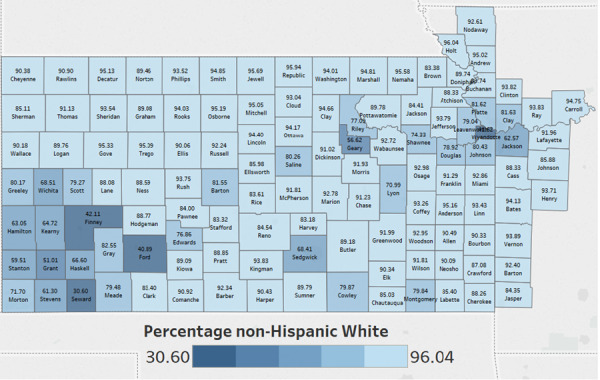
The University of Kansas Cancer Center catchment area counties color coded by the percentage of non-Hispanic White in each county.

**Figure 5:**
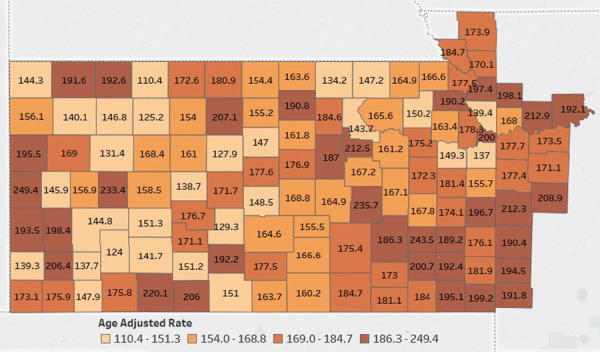
The University of Kansas Cancer Center catchment area counties color coded by the age adjusted cancer mortality rates. Data Source: Centers for Disease Control and Prevention.

**Figure 6:**
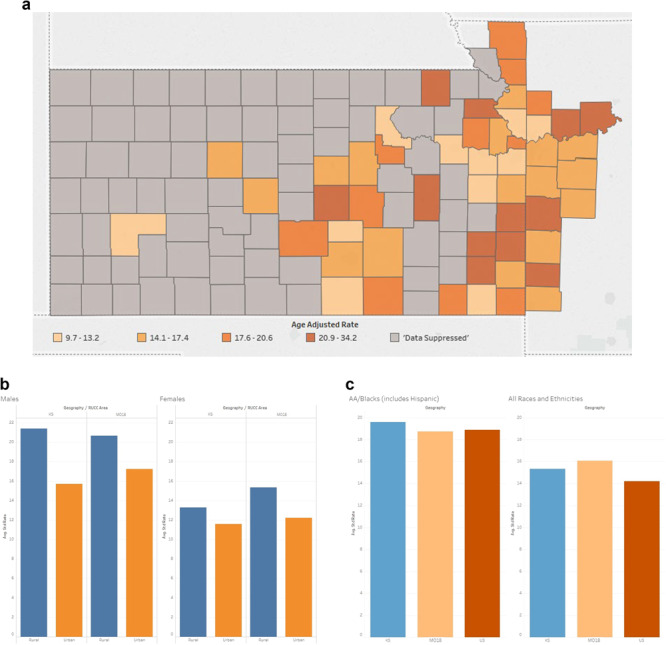
(**A**) The University of Kansas Cancer Center catchment area counties’ heat map representing the mortality rates of colorectal cancer. Colorectal cancer mortality data was suppressed in 72 counties, all of which had populations <34 000. (**B**) The University of Kansas Cancer Center catchment area average standard rate capturing the mortality rates of colorectal cancer comparing rural versus urban. (**C**) The University of Kansas Cancer Center catchment area average standard rate capturing the mortality rates of colorectal cancer comparing African American versus all the other races.

Although the majority population for the KU Cancer Center catchment area is White, there are rural counties in southwest Kansas that are minority–majority, wherein the non-Hispanic White population is below 50% (Figure 4). In addition, counties in rural southeast Kansas have higher levels of poverty, and fewer individuals are insured compared to counties in other areas of the catchment area. This diversity, not only racial and ethnic, but rural versus urban, emphasizes the need for OPTIK, which facilitates a deeper understanding of our communities. For example, data in Figure 5 reveals many counties in our catchment area have higher cancer mortality rates than the national average of 156 per 100 000. OPTIK can layer information about insurance status and cancer disparities to provide additional insight and assist with strategic planning of research and outreach efforts. Furthermore, using OPTIK to map specifically the mortality rates of colorectal cancer in our catchment area reveals some counties have higher rates when compared to Kansas (14.8 (14.2, 15.4)—95% confidence interval) and Missouri state rates (15.1 (14.7, 15.5)—95% confidence interval), as visualized by Figure 6A–C. This data was presented to and reviewed by KU Cancer Center leadership, program leaders, researchers and the community. It was determined that education and screening efforts should focus specifically on Black/African American and rural populations. Research has focused on screening decisions that will improve screening rates. Using this information provided by the OPTIK database, the KU Cancer Center received an administrative supplement to The University of Kansas Cancer Center Support Grant (P30CA168524) to support a community health educator focused on improving colorectal cancer screening and clinical trial participation among the Black/African American populations.

Although much of the data in OPTIK reflects publicly available information, the addition of health system level data and inclusion of data in formats that are not readily available allows the KU Cancer Center to prioritize and evaluate the impact of research and outreach efforts. Data from OPTIK has also been used to communicate catchment area cancer burden to community advisory boards and local non-profit boards, the KU Cancer Center’s external advisory board, annual reports and research funding proposals.

### Dissemination

While describing a catchment area is valuable, a cancer center must share those findings with a larger community of individuals whose scope of practice impacts research and care beyond the center’s network (25). Engaging internal and external stakeholders via seminars, strategic planning sessions and meetings is a crucial part of KU Cancer Center’s mission to make a meaningful impact on the region’s cancer burden (19) by influencing research, policy and outreach efforts. Data organized within OPTIK can be shared with researchers, cancer center leaders, community members and public health leaders in the catchment area to support a bidirectional discussion about disparities, resources and gaps, and guide the priorities of the KU Cancer Center. OPTIK also provides powerful visualizations that present cancer prevention, screening and survivorship needs to community members to help focus future cancer control efforts. By sharing data with community advisory boards, KU Cancer Center engages the community to guide outreach efforts. Data regarding the catchment area is available to individual investigators who request information for the development of funding proposals, presentations, posters and manuscripts. By offering a single source that is easily accessed for visualization of vetted data from multiple sources, researcher and administrative time can be spent on prioritizing and implementing research and outreach efforts. A single data source also assures that communication and proposals regarding the catchment area are consistent across the cancer center.

## Discussion

Synthesizing data to identify trends within a catchment area poses a multitude of challenges to researchers who must standardize data collected at different levels from different sources into a readable visualization. Data collected by OPTIK currently exists at the county, state and zip code levels, and it is the responsibility of the statisticians managing the database to find the appropriate level for the data to be standardized. Manipulating this data is difficult, as information from the tumor registry, for example, is only disseminated at the county level. Catchment areas that include rural communities can present additional challenges to reflect accurate data without compromising patient privacy in areas where certain incidence cases are very small. OPTIK can assimilate information at regional levels or cluster counties based on rurality, creating an opportunity to have a level of understanding around cancer health disparities in the catchment area and where to focus research and interventions to improve health equity. Cancer centers with catchment areas that cross state lines may also be challenged to calculate the age adjusted rates of cancer incidence and mortality. OPTIK is a promising approach to assist with the assimilation of the data, no matter the source.

Despite these challenges, OPTIK played a vital role in supplying data trends and information pertaining to the catchment area to identify research, outreach and policy priorities for KU Cancer Center in several ways. First, it provided key support for the KU Cancer Center’s NCI Community Oncology Research Program (NCORP) Minority Underserved successful grant application (UG1239767) as a rural site. Second, OPTIK played a meaningful role in visualizing regions and populations that have a higher colorectal cancer burden. Researchers, community members and the KU Cancer Center’s Cancer Committee for Commission utilized the colorectal mortality data to have a clearer understanding of the colorectal cancer burden and supported the decision to enhance outreach and research efforts to mitigate colorectal cancer disparities. Finally, OPTIK supported the successful submission of the previously mentioned administrative supplement to The University of Kansas Cancer Center Support Grant (P30CA168524) to improve colorectal cancer screening and clinical trial participation among the Black/African American populations. These specific issues within the Black/African American community were identified by the KU Cancer Center’s catchment area committee as they reviewed the data collated by OPTIK.

Understanding the limitations of the findings from OPTIK is essential to ensure that comprehensive data is available and is not extrapolated in any manner beyond an objective assessment of results. For instance, identifying a risk factor *correlating* to an increase in cancer incidence rate does not necessarily mean it *causes* the increase but can be used to identify areas needed for further investigation. In the near future, data from OPTIK will be utilized to build various univariate and bivariate modeling to generate hypothesis among the cancer risk factors. Educators and members of the public health community must recognize that data correlation is not equivalent to causation, and such a relationship cannot and should not be forged (26). By engaging stakeholders and creating bidirectional communication, the understanding of the cancer burden in the catchment area is enhanced.

An additional limitation with the initiation of OPTIK is access to meaningful cancer control data. Relationships and agreements must be built between the cancer center and with those organizations that store the data. For example, to understand human papillomavirus vaccination rates, the National Immunization Survey is useful at the state level, but access to the state immunization registries would provide county-level detail and a direction for OPTIK in the future. Utilizing the catchment committee’s expertise is essential in identifying appropriate data needs and sources and can play a role in developing partnerships with the organizations that have access to the needed data.

There are a host of future goals for the role of OPTIK in KU Cancer Center cancer trend management. The KU Cancer Center aims to keep growing the OPTIK database and strives to collect more sources that will help develop further nuanced insights into catchment area trends. Along those lines, ensuring the timeliness of data sources is essential for KU Cancer Center to prioritize the relevance of the data collected and its usefulness in reflecting accurate community trends. To assure that OPTIK continues to be a resource for all KU Cancer Center members, the resource needs to be accessible to researchers and cancer center leadership with a simplified, timely and efficient approach for data requests. Keeping patient privacy as the utmost priority in data collection and management is another crucial component in ensuring that OPTIK succeeds in being a database that truly serves the KU Cancer Center community. Finally, streamlining the data collection process and its regulatory obstacles is a core efficiency question KU Cancer Center is actively working to address. Leadership at KU Cancer Center aims to have OPTIK data made available to all KU Cancer Center members via a ShinyR application. The ShinyR application will help researchers select and sort the various data elements that are available under OPTIK. Furthermore, the ShinyR application will be able to provide heat maps for the selected variables along with basic descriptive statistics and a comparison between the KU Cancer Center catchment area average versus national average.

## Conclusion

Data visualizations produced by OPTIK help KU Cancer Center investigators convey trends and metrics identified from the data in a meaningful and efficient manner to supplement health impact in grant applications and inform institution administrators and community leaders (22). Teams are using ShinyR to create publicly available standard reports that will generate more detailed visualizations in Tableau based on specified data parameters (by year, gender, race, etc.). This tool will expand the utility of the database to a broader cohort of KU Cancer Center investigators and heighten the value of OPTIK to the institution.
